# Supplementary material for: Characteristic Findings of Infants with Transient Elevation of Acylcarnitines in Neonatal Screening and Neonatal Weight Loss
Source: Int J Neonatal Screen. 2025 Apr 29;11(2):33. doi: 10.3390/ijns11020033 (PMC12101167; doi:10.3390/ijns11020033)
Supplement: Supplementary file 1 [file IJNS-11-00033-s001.zip › IJNS-3560914-supplementary.pptx]

## Slide 1
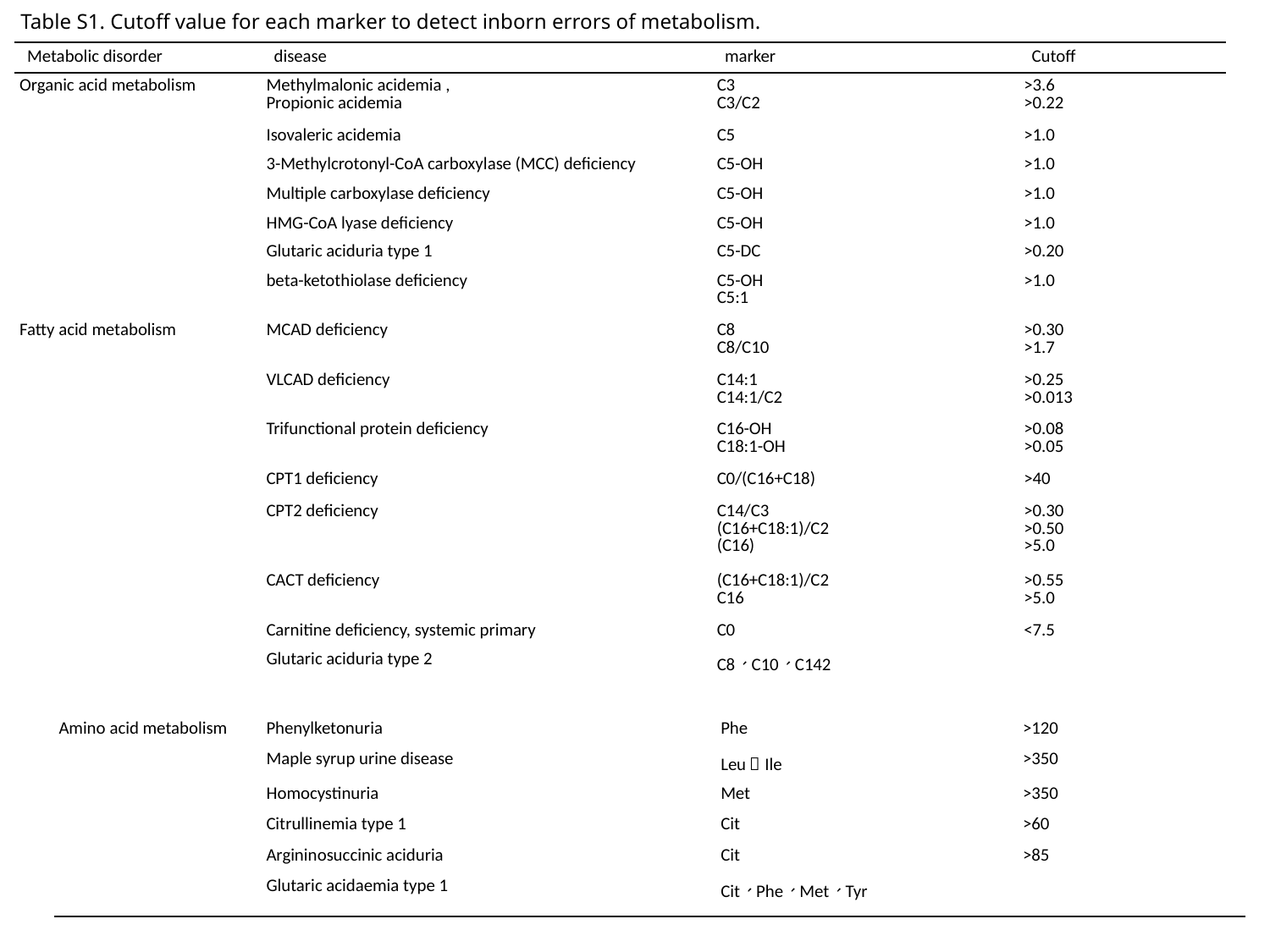

# Table S1. Cutoff value for each marker to detect inborn errors of metabolism.
| Metabolic disorder | disease | marker | Cutoff |
| --- | --- | --- | --- |
| Organic acid metabolism | Methylmalonic acidemia , Propionic acidemia | C3 C3/C2 | >3.6 >0.22 |
| | Isovaleric acidemia | C5 | >1.0 |
| | 3-Methylcrotonyl-CoA carboxylase (MCC) deficiency | C5-OH | >1.0 |
| | Multiple carboxylase deficiency | C5-OH | >1.0 |
| | HMG-CoA lyase deficiency | C5-OH | >1.0 |
| | Glutaric aciduria type 1 | C5-DC | >0.20 |
| | beta-ketothiolase deficiency | C5-OH C5:1 | >1.0 |
| Fatty acid metabolism | MCAD deficiency | C8 C8/C10 | >0.30 >1.7 |
| | VLCAD deficiency | C14:1 C14:1/C2 | >0.25 >0.013 |
| | Trifunctional protein deficiency | C16-OH C18:1-OH | >0.08 >0.05 |
| | CPT1 deficiency | C0/(C16+C18) | >40 |
| | CPT2 deficiency | C14/C3 (C16+C18:1)/C2 (C16) | >0.30 >0.50 >5.0 |
| | CACT deficiency | (C16+C18:1)/C2 C16 | >0.55 >5.0 |
| | Carnitine deficiency, systemic primary | C0 | <7.5 |
| | Glutaric aciduria type 2 | C8、C10、C142 | |
| Amino acid metabolism | Phenylketonuria | Phe | >120 |
| --- | --- | --- | --- |
| | Maple syrup urine disease | Leu＋Ile | >350 |
| | Homocystinuria | Met | >350 |
| | Citrullinemia type 1 | Cit | >60 |
| | Argininosuccinic aciduria | Cit | >85 |
| | Glutaric acidaemia type 1 | Cit、Phe、Met、Tyr | |
